# Prognostic effect of lncRNA SNHG7 on cancer outcome: a meta and bioinformatic analysis

**DOI:** 10.1186/s12885-021-09068-w

**Published:** 2022-01-03

**Authors:** Yunyuan Zhang, Qingwu Tian, Shifeng Huang, Qing Wang, Hongmei Wu, Qian Dong, Xian Chen

**Affiliations:** 1 Department of Clinical Laboratory, the Affiliated Hospital of Qingdao University, Qingdao, 266003 Shandong China; 2 Department of Clinical Laboratory Medicine, the First Affiliated Hospital of Chongqing Medical University, No. 1 Friendship Road, Yuzhong District, Chongqing, 400016 China; 3 Department of Abdominal Ultrasound, the Affiliated Hospital of Qingdao University, Qingdao, 266003 Shandong China; 4 Department of Pediatric Surgery, The Affiliated Hospital of Qingdao University, Qingdao, 266003 Shandong China; 5 Shandong Key Laboratory of Digital Medicine and Computer-Assisted Surgery, Qingdao, 266003 Shandong China; 6 Shandong College Collaborative Innovation Center of Digital Medicine Clinical Treatment and Nutrition Health, Qingdao, 266003 China

**Keywords:** Cancer, SNHG7, meta-analysis, prognostic biomarker

## Abstract

**Background:**

New evidence from clinical and fundamental researches suggests that SNHG7 is involved in the occurrence and development of carcinomas. And the increased levels of SNHG7 are associated with poor prognosis in various kinds of tumors. However, the small sample size was the limitation for the prognostic value of SNHG7 in clinical application. The aim of the present meta-analysis was to conduct a qualitative analysis to explore the prognostic value of SNHG7 in various cancers.

**Methods:**

Articles related to the SNHG7 as a prognostic biomarker for cancer patients, were comprehensive searched in several electronic databases. The enrolled articles were qualified via the preferred reporting items for systematic reviews and meta-analysis of observational studies in epidemiology checklists. Additionally, an online database based on The Cancer Genome Atlas (TCGA) was further used to validate our results.

**Results:**

We analyzed 2418 cancer patients that met the specified criteria. The present research indicated that an elevated SNHG7 expression level was significantly associated with unfavorable overall survival (OS) (HR = 2.45, 95% CI: 2.12–2.85, *p* <0.001). Subgroup analysis showed that high expression levels of SNHG7 were also significantly associated with unfavorable OS in digestive system cancer (HR = 2.31, 95% CI: 1.90–2.80, *p* <0.001) and non-digestive system cancer (HR = 2.67, 95% CI: 2.12–3.37, *p* <0.001). Additionally, increased SNHG7 expression was found to be associated with tumor stage and progression (III/IV vs. I/II: HR = 1.76, 95% CI: 1.57–1.98, *p* <0.001). Furthermore, elevated SNHG7 expression significantly predicted lymph node metastasis (LNM) (HR = 1.98, 95% CI: 1.74–2.26, *p* <0.001) and distant metastasis (DM) (HR = 2.49, 95% CI: 1.88–3.30, *p* <0.001) respectively. No significant heterogeneity was observed among these studies. SNHG7 was significantly upregulated in four cancers and the elevated expression of SNHG7 predicted shorter OS in four cancers, worse DFS in five malignancies and worse PFI in five carcinomas based on the validation using the GEPIA on-line analysis tool.

**Conclusions:**

The present analysis suggests that elevated SNHG7 is significantly associated with unfavorable OS, tumor progression, LNM and DM in various carcinomas, and may be served as a promising biomarker to guide therapy for cancer patients.

**Supplementary Information:**

The online version contains supplementary material available at 10.1186/s12885-021-09068-w.

## Background

With the increasing prevalence of cancer, carcinomas had gradually been recognized as a major threaten to human health the world over [1, 2]. Although great progresses continued to be made in cancer treatment, it is not satisfactory because the long-term survival rate of many cancers is still remaining very low. With the rapid developments of science and technology, researchers gradually realize that the molecular mechanisms of tumorigenesis and development are still need further elucidated. Therefore, there is an urgent need to find new and effective clinical biomarkers for early diagnosis, prognosis and ideal therapeutic targets for cancer patients. Long noncoding RNAs (lncRNAs) have a wide range of biological functions, such as alternative splicing, chromatin modification, dosage compensation, inactivation of major tumor suppressor genes and gene imprinting etc [3–5].

As a modulator of biological processes, small nucleolus RNA host gene 7 (SNHG7) that located on chromosome 9q34.3, has been firstly discovered in lymphoblastoid cell lines. New evidence from clinical and fundamental researches suggests that lncRNA SNHG7 is expressed in many tissues and involved in the occurrence and development of various carcinomas. Researches have suggested that SNHG7 may associated with methylation. For example, Wu et al. had discovered that upregulated DNMT1 could induce hypermethylation of the SNHG7 promotor in hypopharyngeal cancer cells [6]. It was also reported that SNHG7 could directly bind DNMT1, which in turn binds the p53 promoter region, thus inhibiting its expression at the epigenetic level [7]. Recently, SNHG7 was discovered as differentially m^6^A-methylated and expressed lncRNAs in gastric cancer [8]. In cell nucleus, several proteins have been found to be regulated by SNHG7, including Bax and p21, p15 and p16, and β-catenin pathway members [9–12]. Due to the different nature of various cancer types, several signaling molecules associated with SNHG7 has gradually been unveiled, such as AKT2, BCL-2, BCRP, BDNF, CDK6, CTNNB1, Cyclin D1, DNMT1, E-cadherin, ELAVL1, ELK1, EMT, FAIM2, GALNT1, ID4, MDR1, Notch1, p15, p16, p21, PI3K/AKT/mTOR, PVT1, ROCK1, SMAD4, SOX4, SYVN1, TGF-β, WNT2B, Wnt/β-catenin etc [9, 10, 12–29].

Mounting evidences revealed that lncRNAs are deregulated in a variety of carcinomas. Therefore, lncRNAs have attracted extensive attention and may be served as potential biomarkers for carcinomas. Different studies have explored that the increase levels of lncRNA SNHG7 are associated with poor prognosis in various kinds of tumors. However, the small sample size was the limitation for the prognostic value of SNHG7 in clinical application. In the present study, a qualitative meta-analysis was conducted to explore the prognostic effect of SNHG7 on cancer patients.

## Methods

### Literature search and selection

Articles published in English up to Dec 30^th^, 2020, which related to the lncRNA SNHG7 as a prognostic biomarker for cancer patients, were comprehensive searched in several electronic databases. These databases include: Springer, Cochrane Library, Embase, BioMed Central, PubMed, Science Direct and ISI Web of Knowledge. Articles with the following keywords for the online search were included: (“SNHG7” OR “small nuclear RNA host gene 7” OR “lnc RNA-” OR “long noncoding RNA-” OR “noncoding RNA-”) AND (“neoplasm” OR “tumor” OR “cancer” OR “carcinoma”) AND (“metastasis” OR “prognosis” OR “metastatic” OR “prognostic”). Manually searched of the reference lists were also conducted to obtain potential eligible studies.

### Inclusion and exclusion criteria

Inclusion criteria: 1) definite diagnosis or histopathology confirmed for carcinomas;2) studies investigating the prognostic values of lncRNA SNHG7 in various cancers; 3) sufficient information for computing pooled hazard ratios (HR) and 95% confidence intervals (CI).

Exclusion criteria: 1) duplicated articles; 2) studies absent of prognostic outcomes; 3) case reports, correspondences, letters, non-human research, review articles and other studies without original data.

### Data extraction and quality assessment

After reviewed the eligible articles, two authors (YYZ and XC) extracted the necessary data independently. The necessary information from each publication was extracted: (1) last name of first author, publication year, country, cancer type, study design, stage, follow-up time and total cases; (2) SNHG7 assessment method and specimen resources; (3) hazard ratio (HR) with 95% confidence interval (CI) of SNHG7 for overall survival; (4) patient number for TNM stage and progression, lymph node metastasis and distance metastasis. Preferred reporting items for systematic reviews and meta-analyses (PRISMA) was served to qualified all of the enrolled articles (Supplementary Table S1). Enguage Digitizer (Version 4.1) software was performed to extract HRs with 95% CIs from the graphical plots if the eligible literature only provided Kaplan–Meier survival curves as the OS data [30, 31].

### Validation of bioinformatics database

Gene Expression Profiling Interactive Analysis (GEPIA), a web-based tool to deliver fast and customizable functionalities based on TCGA and GTEx data (http://gepia.cancer-pku.cn/). Survival plots of the correlation between SNHG7 expression and OS or DFS were retrieved as K–M curves based on different cancer datasets from GEPIA online database. Progression free interval (PFI) analysis of various carcinomas was based on the transcriptome sequencing data from TCGA. Median was set for cutoff value. Differential expression analysis between cancer and normal tissues was conducted based on GEPIA on-line analysis. All *p*-value < 0.05 was regarded as significantly statistical.

### Statistical analysis

The effect of SNHG7 levels on the aggregated overall survival, tumor progression, lymph node metastasis and distance metastasis were evaluated by HRs and 95% CIs. *I*^2^ statistics was used to calculated heterogeneity among the enrolled studies. The fix-effects model was performed to reveal the relationship between SNHG7 expression levels and clinical outcomes (*I*^2^< 50%) [32, 33]. Probable publication bias was evaluated by a funnel plot and Begg’s bias test [34]. A *p-*value < 0.05 was regarded as significantly statistical. All analyses were conducted with RevMan 5.3 software and Stata SE 12.0 (Stata Corporation).

## Results

### Included articles

Literature screening and study selection processes were presented as Fig. S1. The preliminary online search retrieved 548 publications concerning the prognosis or metastasis of SNHG7 and cancer patients. After carefully removing the duplicates, 28 articles were excluded and 408 publications proceed to abstract screening. We then carefully remove another 385 studies according to the inclusion and exclusion criteria. Finally, 23 articles were enrolled for the meta-analysis in this study.

### Characteristics of the enrolled studies

Table 1 summarized the main characteristics of the enrolled twenty-three eligible articles [6, 10, 18, 20, 21, 24, 25, 35–50]. All of the 2418 participants were from China and divided into high or low group according to the qRT-PCR or microarray results. The cut-off values were different, with median was applied in most articles. Nineteen of the enrolled studies investigated the expression level of SNHG7 and overall survival. Twenty-two articles were associated with the level of SNHG7 and tumor progression or metastasis.

**Table 1:** Summary of the twenty-three included studies

	Study	Origin of population	Study design	Disease	Number	Stage	SNHG7 assay	Survival analysis	Metastasis analysis	Hazard ratios	Follow-up Months
1	Chen 2018 [10]	China	R	Bladder cancer	92	I-III/IV	qRT-PCR	OS	LNM/DM	K-M	60
2	Cheng 2019 [25]	China	R	Pancreatic cancer	40	I-II/III-IV	qRT-PCR	OS	LNM	K-M	50
3	Chi 2018 [49]	China	R	Neuroblastoma	92	I-IIA/IIB-IV	qRT-PCR	OS	LNM	K-M	60
4	Hu 2019 [35]	China	R	Colorectal cancer	738	NA	TCGA database	OS	NA	K-M	14
5	Jia 2020 [36]	China	R	Neuroblastoma	45	I-II/III-IV	qRT-PCR	OS	NA	K-M	60
6	Jiang 2020 [37]	China	R	Breast cancer	57	I-II/III-IV	qRT-PCR	NA	LNM	NA	NA
7	Li 2018 [38]	China	R	Colorectal cancer	70	I-II/III-IV	qRT-PCR	OS/DFS	LNM/DM	K-M	60
8	Pang 2020 [39]	China	R	Non-small cell lung cancer	42	I-II/III-IV	qRT-PCR	NA	LNM	NA	NA
9	Qi 2018 [20]	China	R	Prostate cancer	42	II/III-IV	qRT-PCR	OS	LNM	K-M	60
10	Shan 2018 [24]	China	R	Colorectal cancer	48	I-II/III-IV	qRT-PCR	OS	LNM/DM	K-M	72
11	Shen 2020 [40]	China	R	Hepatocellular carcinoma	100	I-II/III-IV	qRT-PCR	OS/PFS	LNM	K-M	60
12	Wang 2019 [18]	China	R	Thyroid cancer	64	I-II/III-IV	qRT-PCR	NA	LNM	NA	NA
13	Wang 2020 [23, 27, 41]	China	R	Melanoma	80	I-II/III-IV	qRT-PCR	OS	LNM/DM	K-M	48
14	Wu 2019 [6]	China	R	Hypopharyngeal Cancer	73	I-II/III-IV	qRT-PCR	OS	LNM/DM	K-M	60
15	Wu 2020 [42]	China	R	Cervical Cancer	51	I-II/III-IV	qRT-PCR	OS	LNM	K-M	60
16	Xia 2019 [50]	China	R	Prostate cancer	127	I-II/III-IV	qRT-PCR	OS	LNM/DM	HR/K-M	60
17	Xie 2020 [43]	China	R	Hepatocellular carcinoma	80	NA	qRT-PCR	OS	NA	K-M	60
18	Yang 2019 [44]	China	R	Hepatocellular carcinoma	80	NA	qRT-PCR	OS	NA	K-M	60
19	Zeng 2019 [45]	China	R	Cervical cancer	60	I-II/III-IV	qRT-PCR	OS	LNM	HR/K-M	60
20	Zhang 2019 [7]	China	R	Gastric cancer	162	I-II/III-IV	qRT-PCR	OS	LNM/DM	K-M	96
21	Zhang 2020 [28, 46, 47]	China	R	Colorectal cancer	96	I/II-III	qRT-PCR	OS	LNM	K-M	40
22	Zhao 2020 [48]	China	R	Cervical cancer	45	I-II/III-IV	qRT-PCR	OS	LNM	K-M	50
23	Zhong 2018 [21]	China	R	Bladder cancer	134	I/II-IV	qRT-PCR	NA	LNM	NA	36

*BC* breast cancer, *BLC* Bladder cancer, *CC* cervical cancer, *CRC* colorectal cancer, *HCC* hepatocellular carcinoma, *GA* gastric cancer, *PC* prostate cancer, *PAC* Pancreatic cancer, *TC* thyroid carcinoma, *HC* Hypopharyngeal Cancer, *NSCLC* Non-small cell lung cancer

### Meta-analysis results

**Fig. 1:**
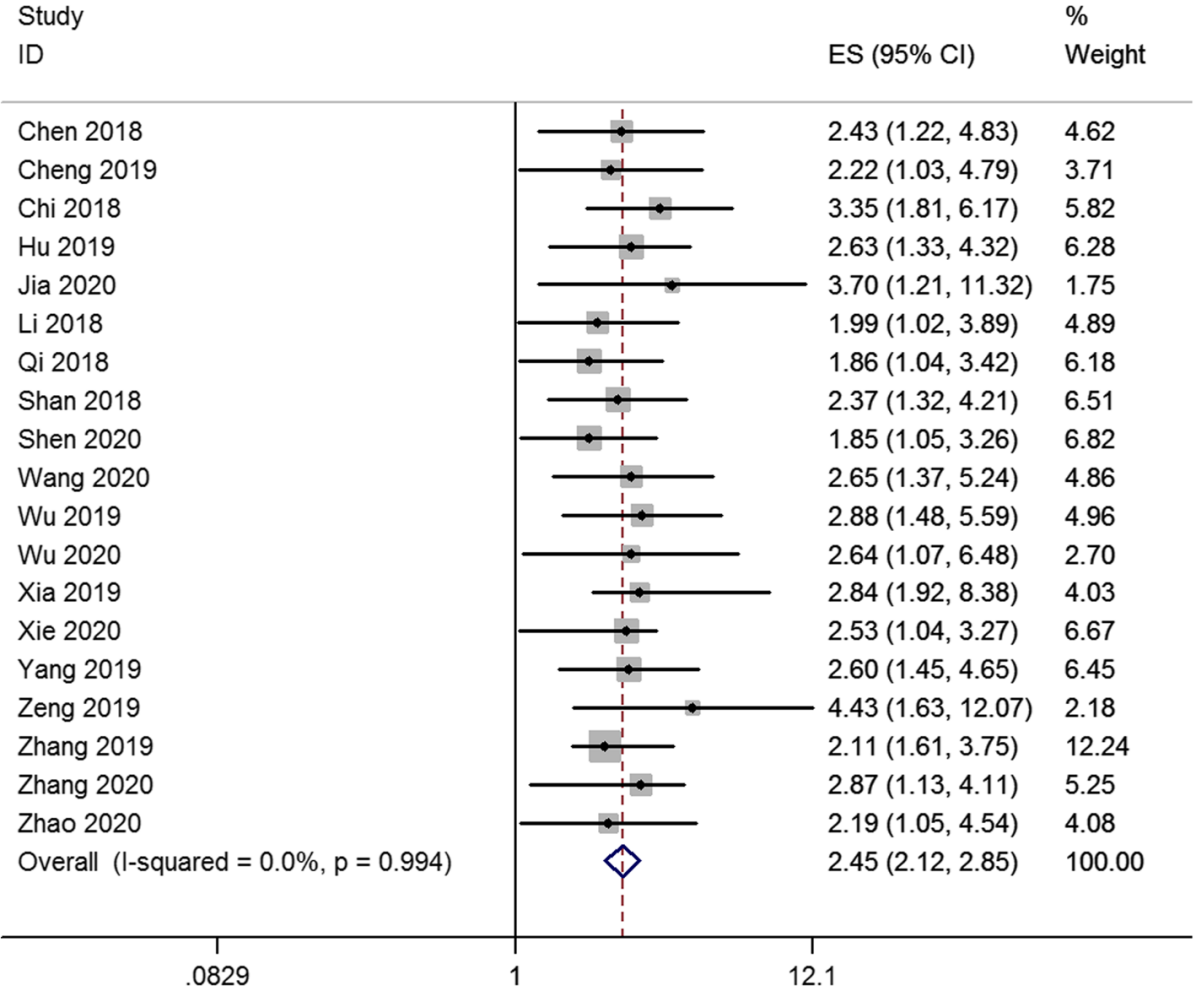
Forest plot of enrolled studies for the association between SNHG7 expression levels with overall survival (OS)

**Fig. 2:**
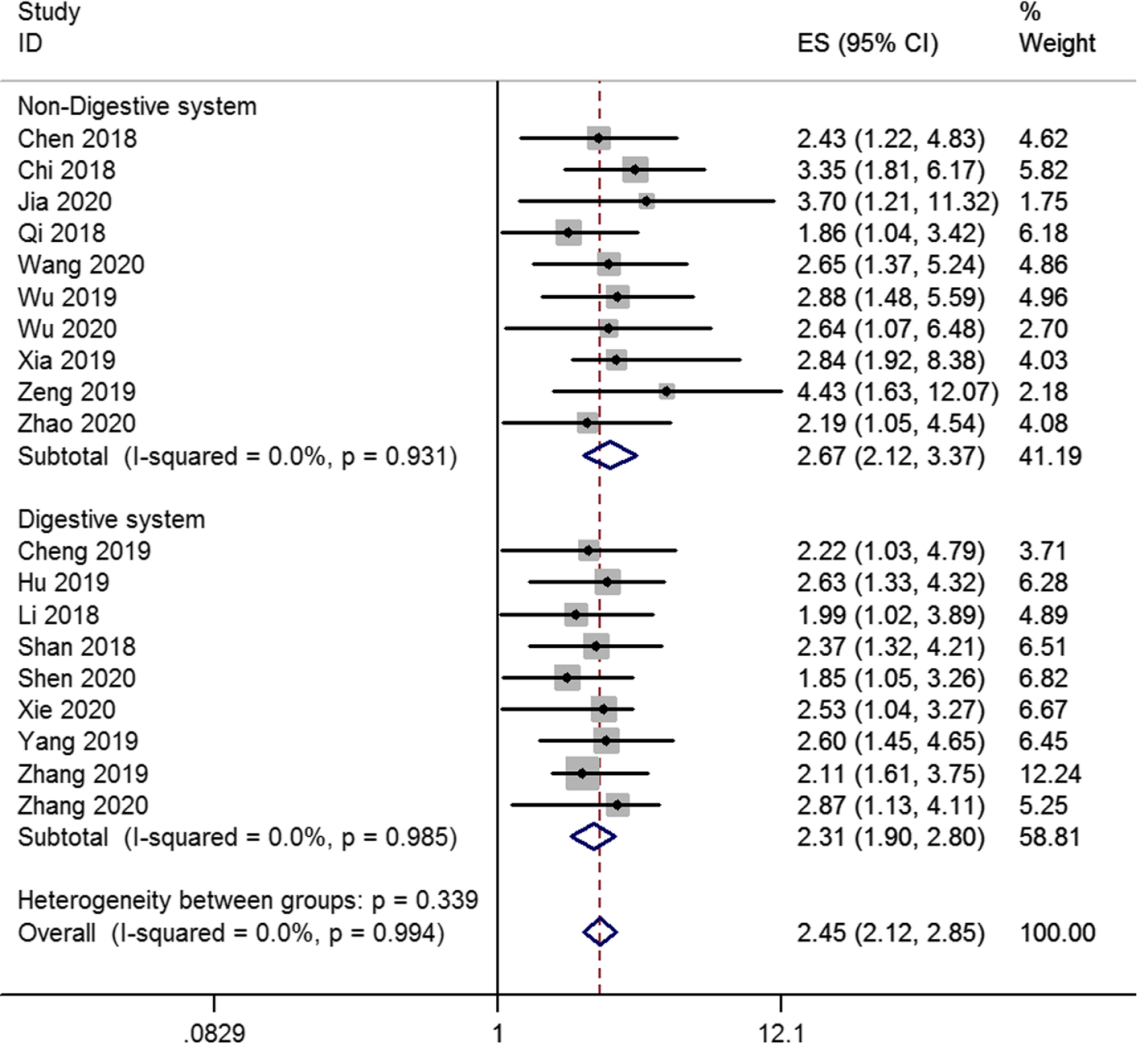
Stratified analysis by factor of cancer type for the association between SNHG7 expression with OS

Finally, nineteen studies were enrolled to analyze lncRNA SNHG7 expression levels and cancer patient outcomes. A fix-effects model was conducted to calculate the pooled effect size because no significant heterogeneity was existed among the enrolled studies (*I*^2^ = 0%). Our results revealed that the increased SNHG7 was significantly related to the unfavorable OS (HR = 2.45, 95% CI: 2.12 – 2.85, *p*<0.001) (Fig. 1). Subgroup analysis showed that high expression levels of SNHG7 were also significantly associated with unfavorable OS in digestive system cancer patients (HR = 2.31, 95% CI: 1.90–2.80, *p*<0.001) and non-digestive system cancer patients (HR = 2.67, 95% CI: 2.12–3.37, *p*<0.001) (Fig. 2).

**Fig. 3:**
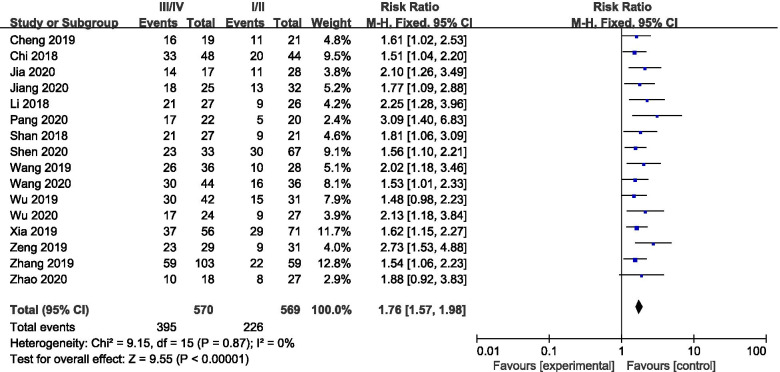
Forest plot of enrolled studies for the association between SNHG7 expression levels with TNM stage (III/IV vs. I/II)

**Fig. 4:**
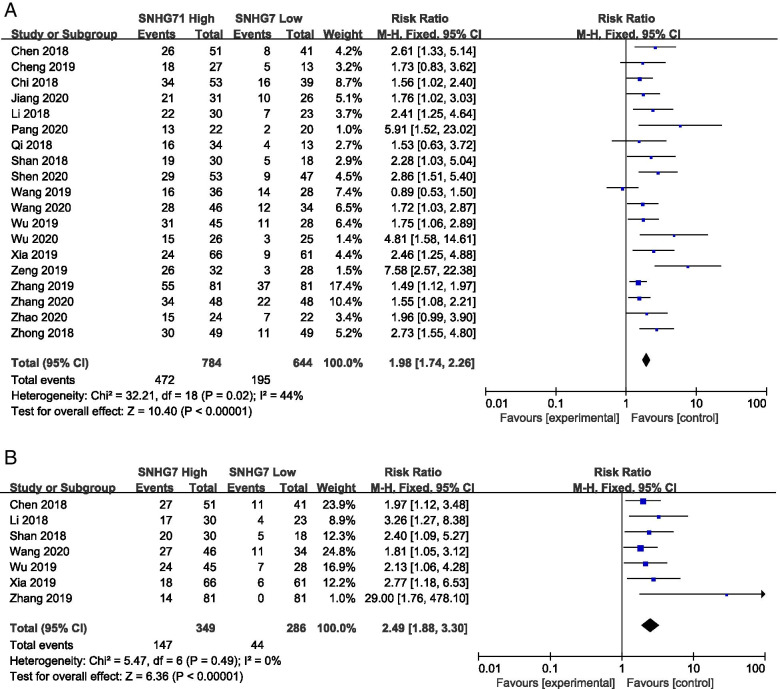
**A** Forest plot for the association between SNHG7 expression levels with LNM. **B** Forest plot for the association between SNHG7 expression levels with DM

Additionally, increased SNHG7 expression was found to be associated with tumor stage and progression (III/IV vs. I/II: HR = 1.76, 95% CI: 1.57–1.98, *p*<0.001) (Fig. 3). Furthermore, elevated SNHG7 expression significantly predicted lymph node metastasis (LNM) (HR = 1.98, 95% CI: 1.74–2.26, *p*<0.001) and distant metastasis (DM) (HR = 2.49, 95% CI: 1.88–3.30, *p*<0.001) respectively (Fig. 4A and B).

### Publication bias

**Fig. 5:**
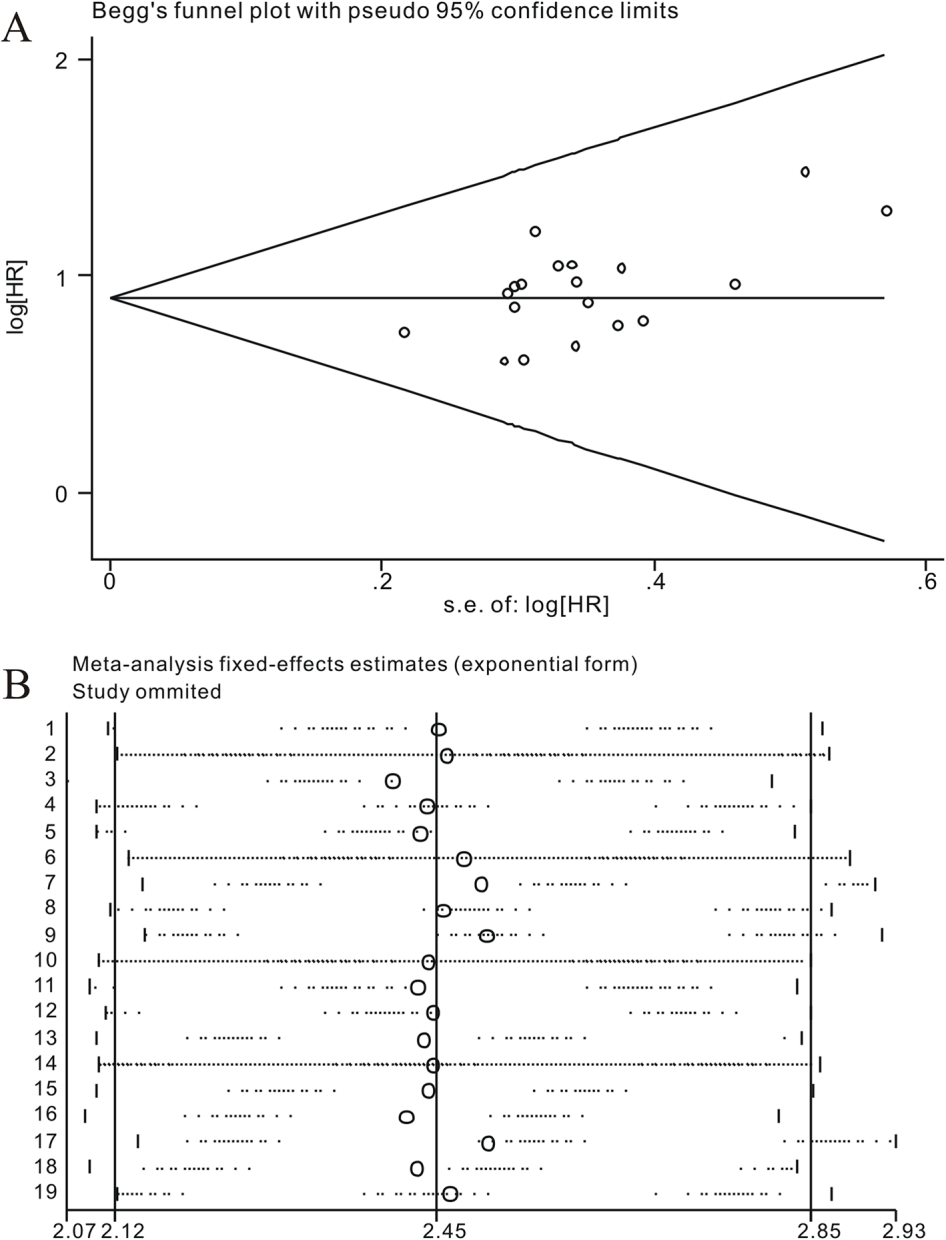
**A** Begg’s funnel plot of publication bias for overall survival. **B** Sensitivity analysis of ten studies concerning SNHG7 and overall survival

Publication bias of the nineteen studies in this analysis was assessed by funnel plot and Begg’s bias test. The shape of the funnel plot was symmetrical and the Begg’s test revealed that no significant publication bias was existed (*p*> 0.05) (Fig. 5A).

### Sensitivity analysis

Through sensitivity analysis of these nineteen enrolled articles, it was indicated that the pooled SNHG7 HR was not significantly affected by the exclusion of any single study (Fig. 5B).

### Validation of the results in the GEPIA database

**Fig. 6:**
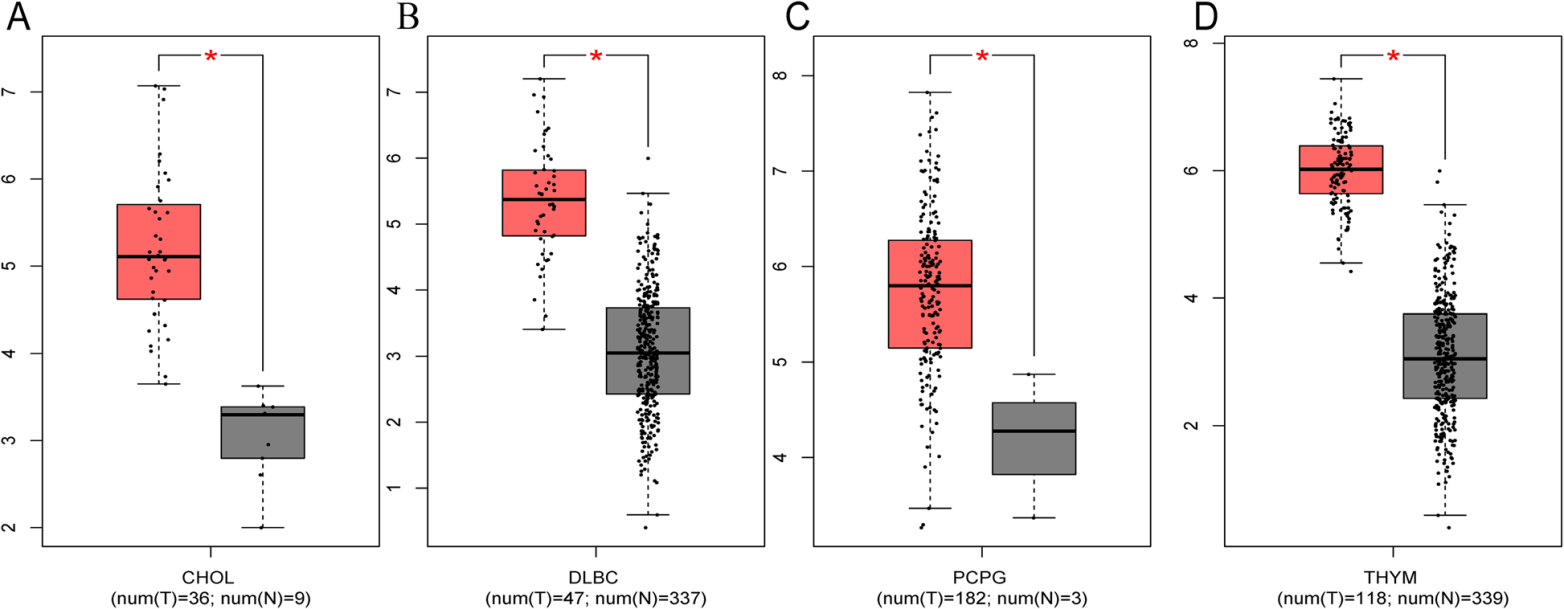
SNHG7 expression in four types of cancer vs. normal tissue. ‘*’ |Log2Fold Change| > 1 and *p* < 0.01. The red box plots represent SNHG7 expression in cancer tissues and the grey box plots represent SNHG7 expression in normal tissues

**Fig. 7:**
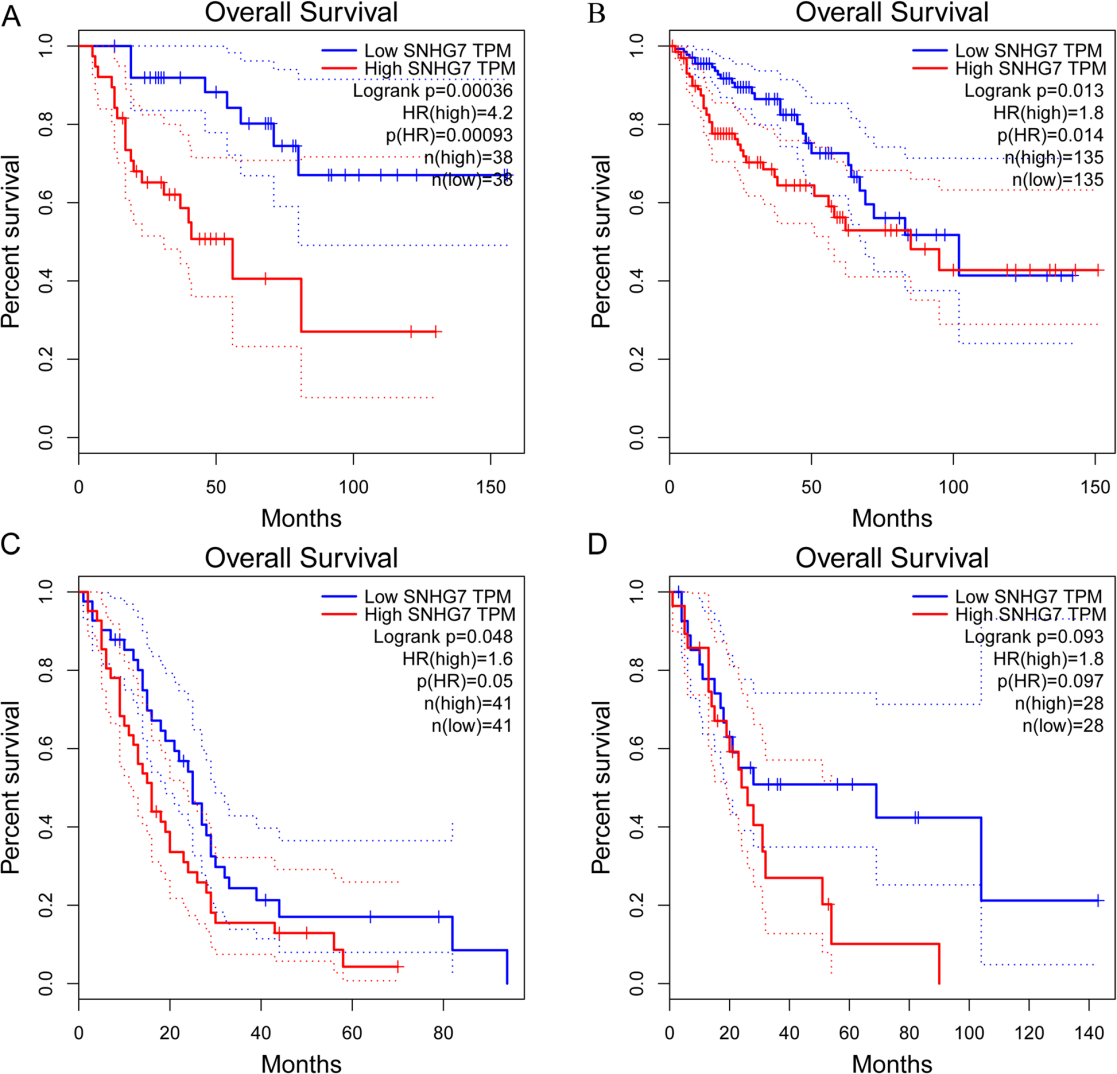
Validation of the prognostic effect of SNHG7 on cancer patient OS based on the GEPIA online database. **A** OS plot of SNHG7 in ACC. **B** OS plot of SNHG7 in COAD. **C** OS plot of SNHG7 in MESO. **D** OS plot of SNHG7 in UCS

**Fig. 8:**
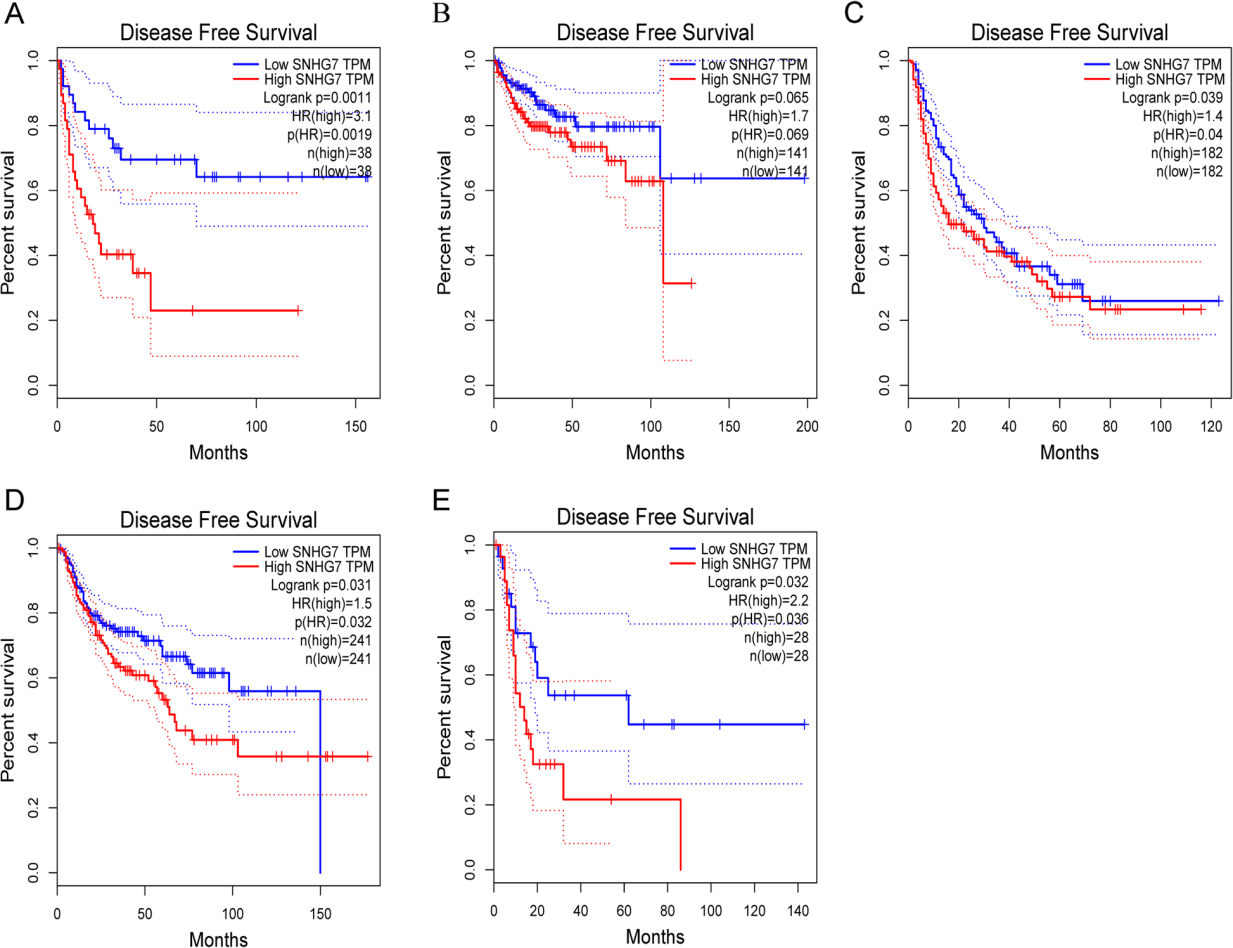
Validation of the prognostic effect of SNHG7 on cancer patient DFS based on the GEPIA online database. **A** DFS plot of SNHG7 in ACC. **B** DFS plot of SNHG7 in KIRP. **C** DFS plot of SNHG7 in LIHC. **D** DFS plot of SNHG7 in LUSC. **E** DFS plot of SNHG7 in UCS

**Fig. 9:**
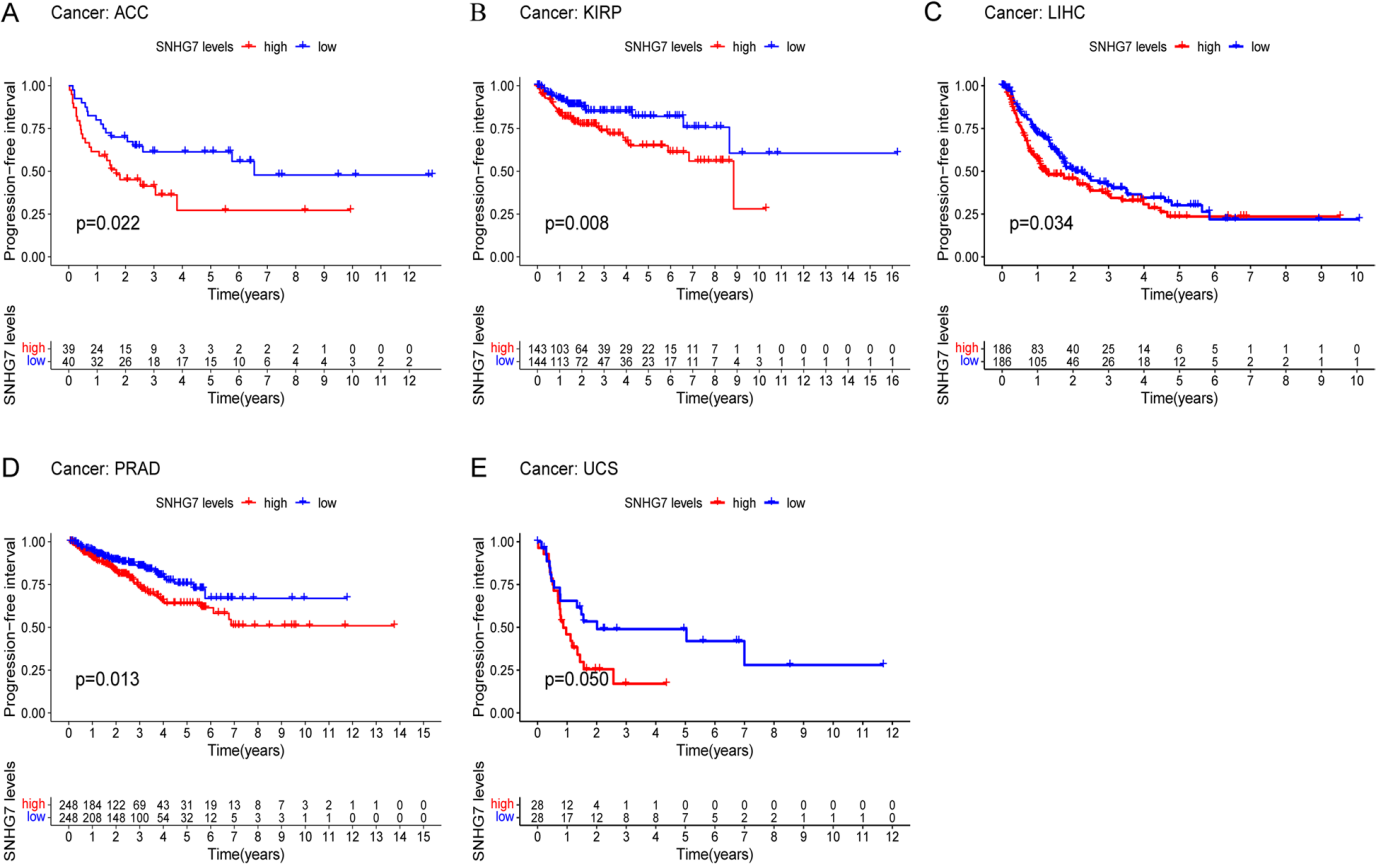
Validation of the prognostic effect of SNHG7 on cancer patient PFI based on the TCGA database. **A** PFI plot of SNHG7 in ACC. **B** PFI plot of SNHG7 in KIRP. **C** PFI plot of SNHG7 in LIHC. **D** PFI plot of SNHG7 in PRAD. **E** PFI plot of SNHG7 in UCS

To further strengthen our conclusion, GEPIA on-line analysis tool was adopted to validate our results (http://gepia.cancer-pku.cn/). In terms of SNHG7 dysregulation, SNHG7 overexpression was identified in Cholangiocarcinoma (CHOL), Lymphoid Neoplasm Diffuse Large B-cell Lymphoma (DLBC), Pheochromocytoma and Paraganglioma (PCPG), and thymoma (THYM) (Fig. 6). Regarding the association between SNHG7 expression and prognosis, increased SNHG7 expression was correlated with worse OS in Adrenocortical carcinoma (ACC), Colon adenocarcinoma (COAD), Mesothelioma (MESO), Uterine Carcinosarcoma (UCS) and with worse DFS in ACC, Kidney renal papillary cell carcinoma (KIRP), Liver hepatocellular carcinoma (LIHC), Lung squamous cell carcinoma (LUSC), UCS. In addition, elevated SNHG7 associated with worse PFI in ACC, KIRP, LIHC, Prostate adenocarcinoma (PRAD) and UCS (*p*<0.05) (Figs. 7, 8 and 9). These results support our results and indicate that SNHG7 could be a novel prognostic biomarker for various cancers.

## Discussion

Early scientists believed that lncRNAs are transcriptional noises due to the fact that most lncRNAs are generated by intron and intergenic regions of the genomes, and lack of protein coding capacity. In recent years, scientists have gradually discovered that lncRNAs may regulate the expression of target genes, involve in biological processes, and may be acted as oncogenes or tumor suppressors. With the rapid expansion of high throughput genomic sequencing technology, lncRNAs have been proved to be deregulated in various tumors, and even to be used as promising prognostic biomarkers in cancer patients. Many clinical and fundamental studies suggested that increasing levels of SNHG7 have intimate terms with unfavorable prognosis and progression in cancer patients. However, the small sample size was the limitation for the prognostic value of SNHG7 in clinical application. As far as we know, no systematic meta-analysis has been performed on SNHG7 expression levels and various cancer patients’ outcomes.

LncRNA SNHG7 has been proved to be significantly up-regulated in various carcinomas, such as bladder cancer, breast cancer, cervical cancer, colorectal cancer, gastric cancer, hepatocellular carcinoma, hypo pharyngeal cancer, melanoma, neuroblastoma, non-small cell lung cancer, pancreatic cancer, prostate cancer, thyroid cancer etc. These aberrant patterns of expression were associated with specific clinical features, such as overall survival time, lymph node metastasis, distant metastasis and TNM stage. SNHG7 serves as an oncogene and contributes to cell biological functions in various cancers, which including apoptosis inhibition, cell proliferation, cell cycle arrest, invasion, migration, and vasculogenic mimicry. Furthermore, SNHG7 may act as a competitive endogenous RNA (ceRNA) to aggravate the development of cancers. Elevated lncRNA SNHG7 may reduce the miRNAs expression level, such as miR-15a, miR-34a, miR-186, miR-193b, miR-216b, miR-342-3p, miRNA-381, miR-503, miR-514a-5p, miR-2682-5p and miR-5095 in multiple cancers [14, 19, 20, 24, 51–54]. Taken together, these articles demonstrated that SNHG7 plays an important role in tumor development and progression. These evidences encouraged us to investigate the correlation between SNHG7 expression levels and cancer prognosis. And our results demonstrated that elevated lncRNA SNHG7 is an unfavorable predictor for various cancer patients.

Twenty-three published studies that included 2418 patients were enrolled in this analysis. Our results revealed that the increased SNHG7 was significantly related to the unfavorable OS (HR = 2.45, 95% CI: 2.12 – 2.85, *p*< 0.001). Subgroup analysis showed that high expression levels of SNHG7 were also significantly associated with unfavorable OS in digestive system cancer (HR = 2.31, 95% CI: 1.90–2.80, *p*<0.001) and non-digestive system cancer (HR = 2.67, 95% CI: 2.12–3.37, *p*<0.001). Additionally, increased SNHG7 expression was found to be associated with tumor stage and progression (III/IV vs. I/II: HR = 1.76, 95% CI: 1.57–1.98, *p*<0.001). Furthermore, elevated SNHG7 expression significantly predicted lymph node metastasis (LNM) (HR = 1.98, 95% CI: 1.74–2.26, *p* <0.001) and distant metastasis (DM) (HR = 2.49, 95% CI: 1.88–3.30, *p*<0.001) respectively. GEPIA and TCGA databases were further used to validate our results as broadly as possible. High SNHG7 expression levels were observed in CHOL, DLBC, PCPG and THYM. What’s more, increased SNHG7 expression was correlated with worse OS in ACC, COAD, MESO, UCS and with worse DFS in ACC, KIRP, LUSC, UCS. In addition, elevated SNHG7 associated with worse PFI in ACC, KIRP, LIHC, PRAD and UCS. Taken together, these results indicate that SNHG7 could be a novel prognostic biomarker for various cancers.

The present meta-analysis has limitations that only the researches published in English were included. Next, this study was constrained to studies published in China, so our results may best illustrate the association between SNHG7 and Asian patients. Well-designed studies and multi-ethnic clinical researches with larger sample size should be carried out in the future. Third, some HRs are extracted by reconstructing the K-M curve, rather than directly from the original research, which would inevitably lead to possible deviations. Despite the inherent deficiencies, our study provides strong evidence that elevated lncRNA SNHG7 expression levels are prognostic for reduced OS, tumor progression, LYM and DM in cancer patients.

## Conclusion

In conclusion, the present analysis implicated that elevated SNHG7 is strongly associated with OS, tumor progression, LNM and DM in carcinomas, and may be served as a promising biomarker to guide therapy for various cancer patients.

## Supplementary Information

**Additional file 1 **: **Figure S1.** Flow diagram of the study search and selection process.

**Additional file 2 **: **Table S1.** PRISMA checklist.

## Data Availability

All data analyzed during this study are included in this published article. GEPIA database is publicly available at http://gepia.cancer-pku.cn/index.html.

## Abbreviations

95% CI: 95% confidence interval
ceRNA: Competing endogenous RNA
PFI: Progression free interval
EFS: Event free survival
HR: Hazard ratio
LncRNA: Long noncoding RNA
OS: Overall survival
PFS: Progress free survival
RFS: Relapse free survival
SNHG7: Small nucleolar RNA host gene 7
