# Supplementary figures and images for: Prognostic effect of lncRNA SNHG7 on cancer outcome: a meta and bioinformatic analysis
Source: BMC Cancer. 2022 Jan 3;22:10. doi: 10.1186/s12885-021-09068-w (PMC8722206; doi:10.1186/s12885-021-09068-w)

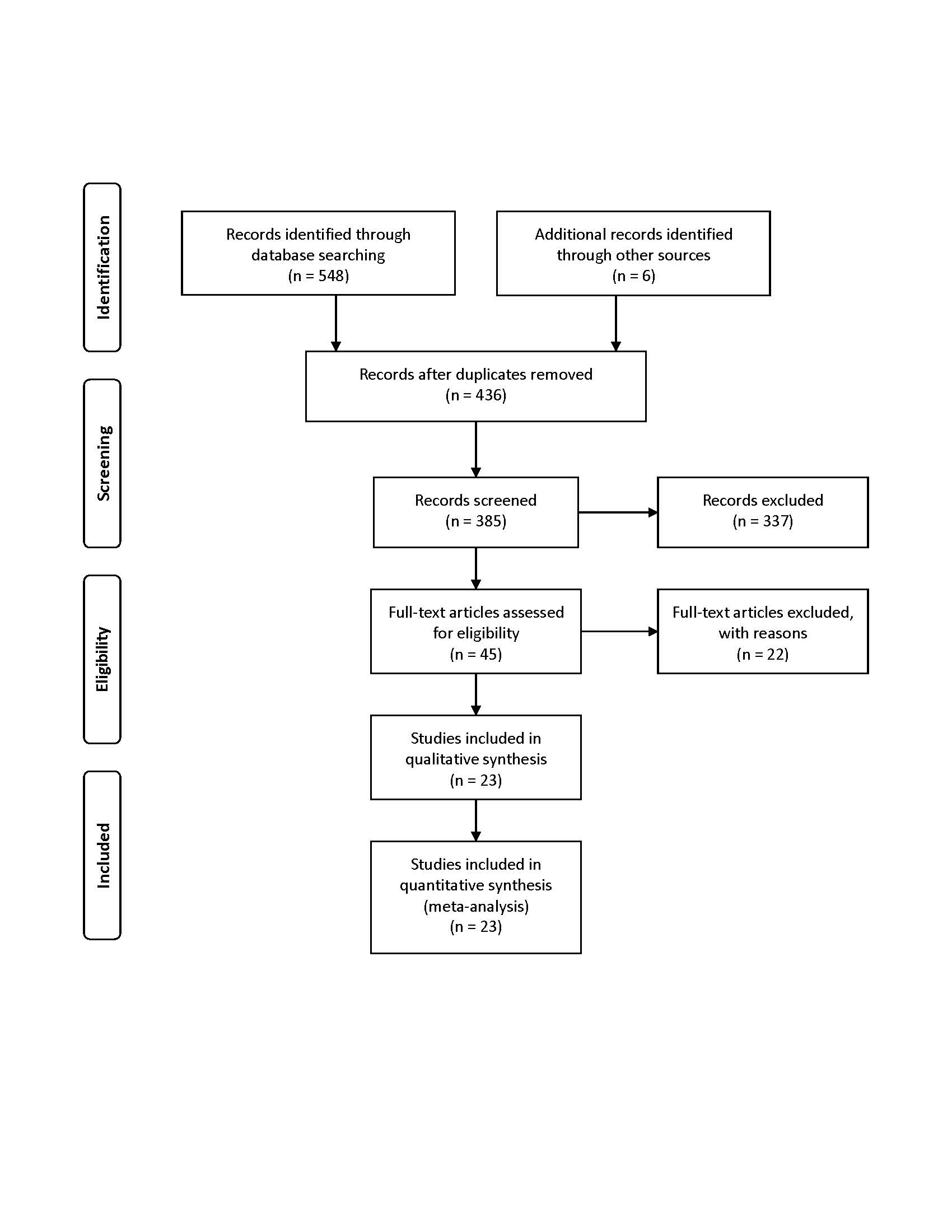

Supplement: Supplementary file 1 — Additional file 1 : Figure S1. Flow diagram of the study search and selection process. [file 12885_2021_9068_MOESM1_ESM.jpg]
